# Microbiological Characterization of Baru Nuts From the Cerrado Biome, Brazil

**DOI:** 10.1111/1750-3841.71043

**Published:** 2026-04-10

**Authors:** Clara Mariana Gonçalves Lima, Dionísio Pedro Amorim‐Neto, Bruna Godoi de Castro, Ana Luísa Perini Leme Giordano, Beatriz Machado Dal Pian, Matheus Péricles Silva Láscaris, Luana Cristina da Silva Ramos, Angélica Zaninelli Schreiber, Luisa Freire Colombo, Anderson S. Sant'Ana

**Affiliations:** ^1^ Department of Food Science and Nutrition Faculty of Food Engineering University of Campinas São Paulo Brazil; ^2^ Department of Pathology Faculty of Medical Sciences University of Campinas São Paulo Brazil; ^3^ Food Technology Department Federal University of Viçosa Minas Gerais Brazil; ^4^ Faculty of Pharmaceutical Sciences, Food and Nutrition Federal University of Mato Grosso do Sul Mato Grosso do Sul Brazil

**Keywords:** *Dipteryx alata Vog*, microbiological quality, microbial ecology, One Health, pathogens

## Abstract

This study aimed to microbiologically characterize baru nuts, a seed widely consumed in Brazil with growing relevance in the export market. Analyses were conducted for mesophilic and thermophilic aerobic spore‐forming bacteria, total mesophilic aerobes, *Enterobacteriaceae*, *Salmonella*, *Staphylococcus aureus*, lactic acid bacteria, molds, and yeasts. Subsequently, Enterobacteriaceae isolates were identified by MALDI‐TOF. It was found that in all baru nut samples, counts of *S. aureus*, lactic acid bacteria, and thermophilic aerobic spore‐forming bacteria were below the quantification limits (< 2log_10_ CFU/g, < 1log_10_ CFU/g, and 1log_10_ spore/g, respectively). All samples had counts above the quantification limits for mesophilic aerobic spore‐forming bacteria (> 1log_10_ CFU/g) and total mesophilic aerobes (> 2log_10_ CFU/g). *Enterobacteriaceae* counts were below 2.22log_10_ CFU/g, while molds and yeasts reached up to 2.70log_10_ CFU/g. Identification of 17 species from the *Enterobacteriaceae* family revealed potentially pathogenic microorganisms that serve as important biological markers, including emerging and multidrug‐resistant species. *Franconibacter pulveris* was the most abundant, representing 41.9% of all isolates, followed by *Pseudescherichia vulneris* and *Pantoea septica*, each accounting for 10.5%. In addition, *Cronobacter* sp. (3.8%) and *Salmonella* sp. (1.0%) were identified—both pathogens commonly associated with nuts and low‐water‐activity products. The presence of these sanitation‐relevant and understudied species highlights the need for ongoing microbiological monitoring and a better understanding of the microbial ecology in food products.

**Practical Applications:**

This study provides information on the microorganisms found in baru nuts. These results can help producers improve handling and processing practices, contributing to safer products for consumers and supporting the development of quality standards for this native Brazilian nut.

## 1 Introduction

Plant extraction carried out by rural families is a highly diverse practice that plays a vital role in Brazil's development. It involves key aspects such as access to and management of natural resources, food acquisition, marketing, and income distribution. Once considered a subsistence activity, it has evolved into an important source of financial income while simultaneously contributing to the conservation of agrobiodiversity (Soares et al. 2018). In this context, the collection and processing of native nuts support thousands of families engaged in agroecological practices across rural regions in several Brazilian states. The activity demands low capital investment and minimal technological input, relying primarily on manual labor for the extraction, transportation, and processing of the product.

The Cerrado is the second largest biome in Brazil, characterized by climatic and soil conditions similar to those of savannas. Its remarkable plant diversity and high level of endemism are attributed to the heterogeneity of habitats and the variability of soil conditions. In this sense, baru (*Dipteryx alata* Vog.) is a native tree species of the Cerrado that flowers from November to May and bears fruit from July to October (Lemos et al. 2012). The fruit consists of a thin outer shell, a fibrous pulp, and a hard woody endocarp that encloses the nut, which is covered by a thin skin. The baru nut is notable for its nutritional and functional properties, as well as its significant commercial value (Alves‐Santos et al. 2021).

Within the production chain of various types of nuts, several stages are vulnerable to contamination due to environmental factors, poor harvesting practices, inadequate post‐harvest handling, improper packaging, and insufficient storage and transportation conditions. Nuts are particularly associated with a high risk of transmitting pathogens such as bacteria and fungi, as well as mycotoxins. Therefore, increased attention and the implementation of appropriate practices both before and after harvest are essential to enhance microbiological safety and ensure shelf‐life stability (Mir et al. 2023). Cross‐contamination can occur at various stages, including the pre‐ and post‐harvest periods, as well as during the handling of the nuts. It is common for small‐scale producers to sell baru nuts to cooperatives and companies, which then take responsibility for the subsequent processing stages. Members of the family *Enterobacteriaceae*, including genera such as *Enterobacter*, *Klebsiella*, *Citrobacter*, and *Cronobacter*, may be found in food production environments and may serve as indicators of hygiene conditions and potential contamination during processing. Some members of this family are also recognized as opportunistic or emerging pathogens of public health concern. Previous studies have reported the presence of *Salmonella* in different types of nuts, highlighting the ability of this pathogen to survive for extended periods in low‐water‐activity foods and representing a relevant food safety concern (Zhang et al. 2021, 2017; Munck et al. 2020; Hu et al. 2023).

In addition to microbiological hazards, low‐water‐activity foods such as edible nuts may also present other food safety risks, including chemical contaminants and processing‐related hazards. Studies conducted in different nut production systems have reported the occurrence of mycotoxins, particularly aflatoxins produced by species of *Aspergillus* (Daou et al. 2023; Musangi et al. 2024), as well as the presence of environmental contaminants such as heavy metals (Xie et al. 2024; Kulluk et al. 2023) and pesticide residues (Onwujiogu et al. 2022; Elmi et al. 2024). These hazards may arise from environmental exposure, agricultural practices, or inadequate post‐harvest handling and storage conditions. Therefore, food safety assessments of nut products should consider the coexistence of microbiological and chemical risks to provide a more comprehensive evaluation of potential threats to consumer health.

As reported by (Carvalho et al. 2025), Brazil is currently the world's largest producer and exporter of baru nuts, with over 50% of its production destined for international markets. In this context, conducting studies focused on monitoring product quality is essential to ensure safety, traceability, and market competitiveness. It is important to emphasize that cooperatives and companies play a key role in strengthening Brazilian agroextractivism. They provide visibility to local communities, preserve cultural traditions, support a stable agricultural workforce, ensure the efficient functioning of the production chain, promote environmental conservation, enhance product competitiveness in the market, and facilitate commercial transactions. Therefore, this study aimed to perform a microbiological characterization of baru nut samples sourced from Brazilian small‐scale agroextractivism using culture‐dependent methods, including species‐level identification of *Enterobacteriaceae* isolates by matrix‐assisted laser desorption/ionization time‐of‐flight mass spectrometry (MALDI‐TOF MS).

## 2 Materials and Methods

### 2.1 Sample Collection

A total of 70 baru nut samples were collected from the Cerrado region in Brazil, specifically from the states of Goiás (48), Minas Gerais (12), Mato Grosso (5), and Mato Grosso do Sul (4), and were obtained from different local producers. All samples were wrapped in kraft paper and stored at room temperature (±25°C) until analysis.

### 2.2 Physicochemical Analysis

#### 2.2.1 pH and Water Activity (aw)

For the analyses, the baru nuts were crushed using a hammer. Then, 10 grams of each sample were homogenized in 90 mL of distilled water, and the pH was measured with a calibrated pH meter (Model K39–2014B, KASVI, China). The water activity (aw) of the samples was determined using an AquaLab 195 moisture analyzer (Model CX‐2, Decagon Devices, Washington, USA). All analyses were performed in triplicate, and the final values were expressed as the mean (IAL, 2005).

### 2.3 Microbiological Analysis

The samples were first crushed with a hammer (Nascimento et al. 2018) and weighed in sterile bags. They were then transferred to sterile glass vials, mixed with the appropriate diluents, and homogenized in an orbital shaker at 250 RPM for 10 min. Afterward, the samples were left to rest for 50 min to facilitate the recovery of stressed cells (ISO 6887‐4, 2017) before undergoing the decimal dilution process.

#### 2.3.1 Total Aerobic Mesophiles

In sterile glass flasks, 25 g of each sample was homogenized with 225 mL of 0.1% peptone water (Oxoid Unipath Ltd., Basingstoke, Hampshire, United Kingdom). Enumeration was performed using the serial dilution technique, followed by surface plating on plate count agar (PCA) (KASVI, Pinhais, Brazil). The plates were incubated at 35°C for 48 h (Ryser and Schuman 2015), and the results were expressed as the total aerobic mesophilic count per gram of sample (CFU/g).

#### 2.3.2 Lactic Acid Bacteria

Briefly, 25 g of the sample was homogenized in 225 mL of peptone water (0.1% w/v), and different serial dilutions were plated by depth using de Man, Rogosa, and Sharpe (MRS) Agar (Oxoid Unipath Ltd., Basingstoke, Hampshire, United Kingdom). Once the plates had solidified, a second layer of medium was added to promote microaerophilia. The plates were incubated at 35°C for 72 h. Confirmation of lactic acid bacteria was performed by isolating representative colonies, which were subjected to Gram staining and a catalase test. Because the matrix under study is of plant origin, the culture medium was acidified with sterile glacial acetic acid until the pH reached 5.5 to inhibit the growth of sporogenic bacteria (ISO, 1998).

#### 2.3.3 Filamentous Fungi and Yeasts

In sterile glass flasks, 25 g of each sample was homogenized with 225 mL of 0.1% peptone water (Oxoid Unipath Ltd., Basingstoke, Hampshire, United Kingdom). Enumeration was performed using the serial dilution technique, followed by surface plating on Dichloran Glycerol (DG‐18) Agar (Merck, Darmstadt, Germany). The plates were incubated at 25°C for 5 days (Ryu and Wolf‐Hall 2015). The results were expressed as the total count of fungi and yeasts per gram of sample (CFU/g).

#### 2.3.4 *Salmonella* sp

Briefly, 25 g of each sample was homogenized with 225 mL of buffered peptone water (BPW) and incubated at 37 ± 1°C for 18 ± 2 h. This was followed by selective enrichment in Rappaport–Vassiliadis soya peptone broth (RVS) at 41.5 ± 1°C for 24 ± 3 h, and in Muller–Kauffmann tetrathionate/novobiocin (MKTTn) broth at 37 ± 1°C for 24 ± 3 h. Both broths were plated onto Xylose Lysine Deoxycholate (XLD) Agar (Merck, Darmstadt, Germany) and *Salmonella Shigella* (SS) Agar (Merck, Darmstadt, Germany), and incubated at 37 ± 1°C for 24 ± 3 h (ISO, 2017a).

#### 2.3.5 Enterobacteriaceae

In sterile glass flasks, 25 g of each sample was homogenized with 225 mL of 0.1% peptone water (Oxoid Unipath Ltd., Basingstoke, Hampshire, United Kingdom). Enumeration of *Enterobacteriaceae* was performed using the serial dilution technique, followed by deep plating on Violet Red Bile Glucose (VRBG) Agar (Merck, Darmstadt, Germany). The plates were incubated at 37°C for 24 h (ISO 21528‐2, 2017). After this period, typical colonies were counted and expressed as CFU/g.

#### 2.3.6 Staphylococcus aureus

In sterile glass flasks, 25 g of each sample was homogenized with 225 mL of 0.1% peptone water (Oxoid Unipath Ltd., Basingstoke, Hampshire, United Kingdom). *Staphylococcus aureus* was enumerated using the serial dilution technique, followed by surface plating on Baird–Parker agar supplemented with egg yolk and potassium tellurite (Merck, Darmstadt, Germany). The plates were incubated at 35°C for 48 h, and presumptive colonies were confirmed by the coagulase test (ISO 6888‐1, 2017).

#### 2.3.7 Counting of Mesophilic Aerobic Sporulating Microorganisms

To enumerate spore‐forming aerobic mesophilic bacteria, the method described by Stevenson and Lembke (2015) was used. In sterile glass flasks, 50 g of baru nuts were combined with 450 mL of peptone water. Aliquots of 10, 1, and 0.1 mL were transferred to three sterile bags, each containing 100 mL of Beef Tryptone Glucose (TGE) Agar, which had been previously melted and cooled to 50°C–55°C. The samples were placed in a water bath at 80°C for 10 min, then cooled to room temperature in water. The medium was subsequently poured into five Petri dishes, which were incubated at 35°C for 48 h. Colonies that developed on the appropriate dilution plates were counted, and the result was expressed as the number of spores per gram (No. of spores/g).

#### 2.3.8 Counting of Aerobic Thermophilic Sporulating Microorganisms

The quantification of spore‐forming aerobic thermophilic bacteria followed the methodology of Olson and Sorrells (2015). In sterile glass flasks, 20 g of baru nuts were added to 100 mL of sterile distilled water and homogenized. Then, 10 mL of the dilution was transferred to a sterile bag containing 100 mL of Dextrose Tryptone Agar (DTA), which was boiled in a water bath for 30 min and subsequently cooled in an ice bath. The medium was then poured into five Petri dishes, which were incubated at 55 *Penicillium*°C for 72 h.

### 2.4 Identification of Enterobacteriaceae Family by Matrix‐Assisted Laser Desorption/Ionization Time‐of‐Flight Mass Spectrometry

Pure colonies of each strain were grown under optimal conditions for species identification by MALDI‐TOF MS, following the manufacturer's recommended direct smear extraction protocol. Briefly, a single colony was transferred to a MALDI target plate (MSP 96 polished steel BC, Bruker Daltonics, Germany) using a sterile toothpick. For protein extraction, 1 µL of 70% formic acid was added directly to each sample spot and allowed to air dry at room temperature. Subsequently, 1 µL of *α*‐cyano‐4‐hydroxycinnamic acid (CHCA) matrix solution (Bruker Daltonics) was applied to the dried spot. Mass spectra were acquired in positive linear mode using a Microflex LT mass spectrometer (Bruker Daltonics, Bremen, Germany). Acquisition parameters included ion source voltages of 2.0 and 1.8 kV, a lens voltage of 6.0 kV, and a laser frequency of 60 Hz, with a mass range of 2000–20,000 Da. The Biotyper software (Bruker Daltonics) was used to compare the acquired spectra with the integrated database (version 3.1). Species identification was performed according to the manufacturer's recommended criteria, where log score values ≥ 2.0 were considered reliable for species‐level determination. External calibration was conducted using the bacterial test standard (Bruker Daltonics, Bremen, Germany).

### 2.5 Statistical Analysis

The statistical analysis of the data was conducted using the Statistical Analysis System (SAS OnDemand) software. Correlation analysis was performed using RStudio software, Version 2024.09.1+394.

## 3 Results and Discussion

**FIGURE 1:**
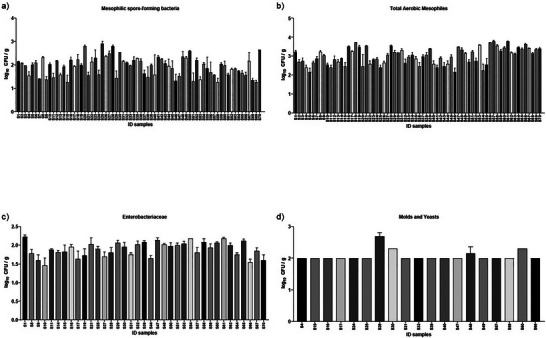
Quantification of microorganisms in baru nut samples by classical microbiological methods. (a) Mesophilic aerobic spore‐forming bacteria; (b) total aerobic mesophiles; (c) Enterobacteriaceae; and (d) filamentous fungi and yeasts. Data are expressed as mean values (log₁₀ CFU/g or log₁₀ spores/g, as appropriate).

**FIGURE 2:**
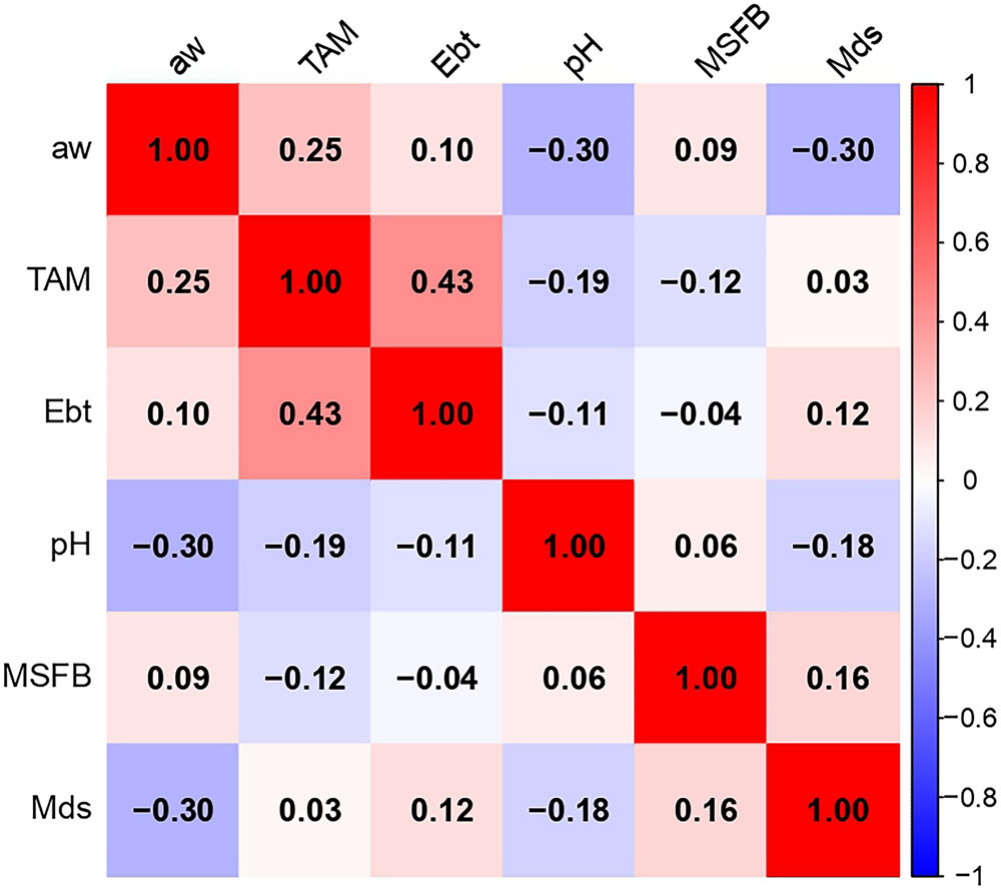
Heat map showing the correlation between aw and pH with mesophilic spore‐forming bacteria (MSFB), total aerobic mesophiles (TAM), *Enterobacteriaceae* (Ebt), and fungi and yeasts (Mds).

It was observed that in all baru nut samples, the counts of *S. aureus*, lactic acid bacteria, and thermophilic aerobic spore‐forming bacteria were below the quantification limits: < 2log_10_ CFU/g, < 1log_10_ CFU/g, and 1log_10_ spore/g, respectively. Figure 1 presents the mean counts for mesophilic aerobic spore‐forming bacteria (a), total aerobic mesophiles (b), *Enterobacteriaceae* (c), and filamentous fungi and yeasts (d). Figure 2 indicates that no correlations were found between the physicochemical data and microbial counts exceeding the quantification limits.

All samples had counts above the quantification limit for mesophilic aerobic spore‐forming bacteria (> 1log_10_ CFU/g) and total mesophilic aerobes (> 2log_10_ CFU/g). *Enterobacteriaceae* counts remained below 2.22log_10_ CFU/g, while molds and yeasts reached up to 2.70log_10_ CFU/g. Carvalho et al. (2025) evaluated fungal contamination and mycotoxins in commercial baru nuts available in the Brazilian market using a validated miniaturized QuEChERS–UPLC–MS/MS method for mycotoxin determination. The fungal isolates were predominantly identified as *Penicillium* spp., and mycotoxin analysis detected alternariol in 8.5% of the samples, with concentrations ranging from 12.2 to 148.2 µg kg^−^^1^. Fumonisins B_1_ (242.7 µg kg^−^^1^) and B_2_ (27.6 µg kg^−^^1^) were detected in one sample (1.4%), while no other mycotoxins, including regulated aflatoxins, were found. In addition, none of the isolated fungal strains showed mycotoxigenic potential. It is important to note that the absence of a rapid and straightforward diagnostic tool for detection, along with limited knowledge of their prevalence and diversity, remains a significant challenge in controlling these microorganisms in food (Postollec et al. 2012). Across different nut production chains, contamination can occur due to environmental factors, improper harvesting practices, inadequate post‐harvest handling, and insufficient packaging, storage, and transportation procedures (Santillana Farakos et al. 2019). A comprehensive understanding of the quantitative microbial ecology of edible nuts is still evolving and is crucial for assessing their safety and overall quality, ultimately impacting their potential shelf life (Fay et al. 2021)

Species within the *Enterobacteriaceae* family are ubiquitous, colonizing humans, animals, insects, plants, water, and soil. These bacteria serve as indicators of hygiene conditions in manufacturing processes, as they are easily inactivated by sanitizers yet capable of colonizing various niches within processing plants when proper sanitization is lacking. Although they cannot multiply in low aw plant products, such as nuts, they can remain viable in these foods for extended periods (Berthold‐Pluta et al. 2021). The *Enterobacteriaceae* family comprises 33 genera and 140 validly published and correctly named species (Moxley et al. 2022)

**FIGURE 3:**
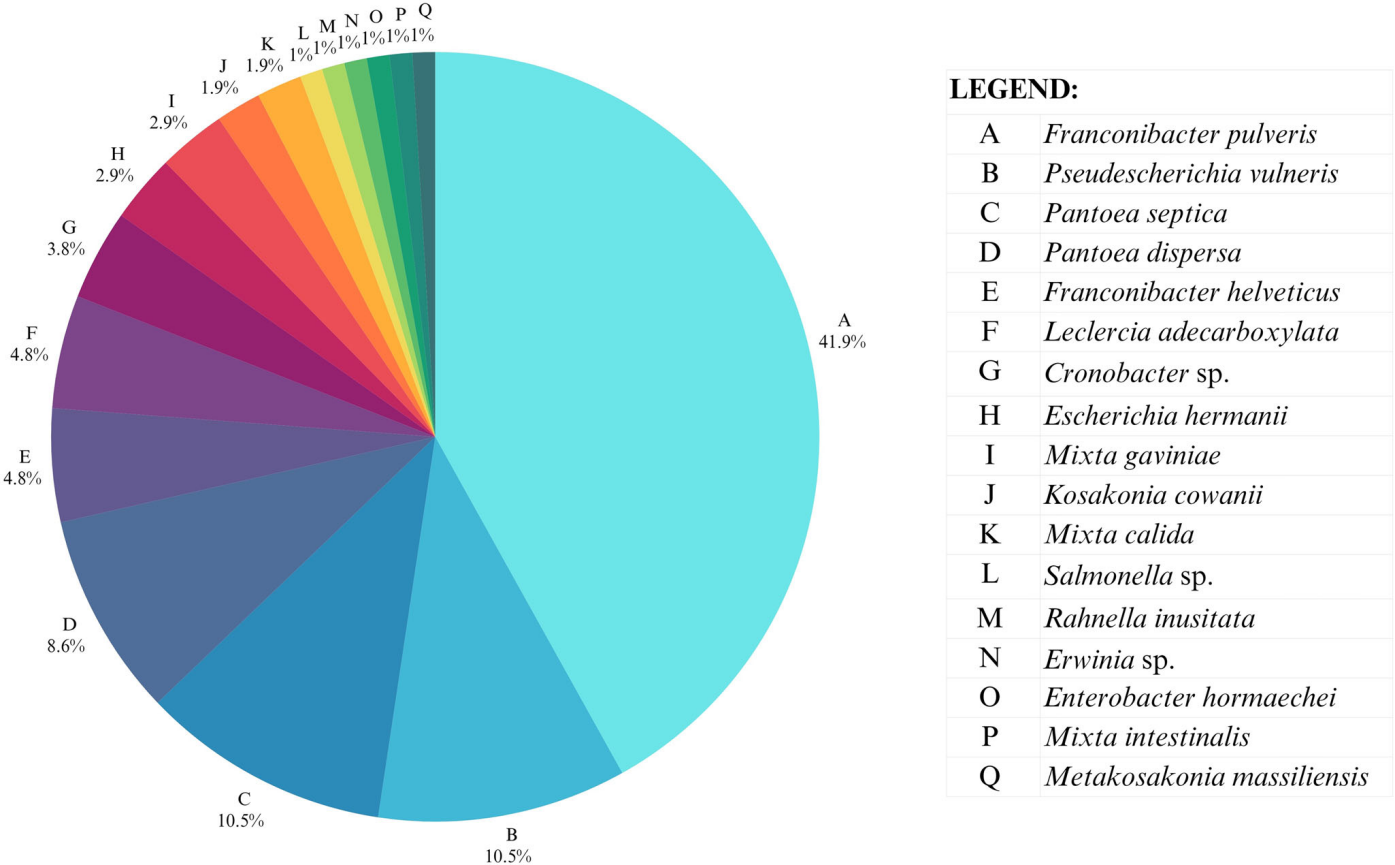
Occurrence frequency of microorganisms identified from isolation on VRBG medium.

The identification of 105 isolates from the *Enterobacteriaceae* family revealed the presence of 17 species, as shown in Figure 3. The most abundant species were: *Franconibacter pulveris*, *Pseudescherichia vulneris*, *Pantoea septica*, *Pantoea dispersa*, and *Franconibacter helveticus*.

Regarding the genus *Franconibacter*, the most recurrent in this study, it is known to be genetically close to the pathogenic genus *Cronobacter* and can be misidentified, sometimes referred to as “pseudo‐*Cronobacter*” (Svobodová et al. 2017). The literature reports the isolation of *Franconibacter* species from low aw products such as herbs, spices, powdered fruits, and infant formulas. There is no indication that *F. pulveris* and *F. helveticus* pose a public health threat (Stephan et al. 2014). Furthermore, these species have not been associated with foodborne outbreaks or infections of public health relevance, and their ecological role remains largely unexplored. However, due to their taxonomic proximity to *Cronobacter*, their detection in food products warrants careful monitoring. *P. vulneris* was established following the taxonomic reclassification of *Escherichia vulneris*, based on phylogenomic and phenotypic analyses. It is recognized as a pathogen of clinical relevance, having been associated with a range of infections, including complicated diarrhea, sepsis, septic arthritis, keratitis, and wound infections (Morales‐López et al. 2019).

The genus *Pantoea* comprises 20 different species, including *P. septica* and *P. dispersa*, which are found in a wide range of natural environments such as soil, water, and plants (Yang et al. 2023). Regarding *P. septica*, the literature reports its isolation from human feces (Brady et al. 2010) and skin (Lo et al. 2015). *P. dispersa* demonstrates tolerance to a wide range of environmental conditions, including elevated salinity and temperature extremes. Although it has been associated with clinical conditions such as respiratory tract infections, neonatal sepsis, and bacteremia, its occurrence as a causative agent in hospital environments remains uncommon (Asai et al. 2019).

*Leclercia adecarboxylata* is found in various environments, including food, and is generally considered harmless. Although traditionally classified as a pathogen of clinical relevance, it can cause a range of infections, particularly bloodstream and urinary tract infections, especially in immunocompromised patients. Recently, it has been recognized as an emerging pathogen capable of infecting immunocompetent individuals. Most infections respond well to common antimicrobial treatments; however, multidrug‐resistant (MDR) strains have been reported worldwide. This makes the species an important reservoir of resistance genes that can be transferred within and between bacterial species, representing a challenge for infection control (Yescas‐Zazueta et al. 2024).

*Cronobacter*, the fifth most prevalent genus identified in the samples, is associated with foodborne diseases (FBDs) classified by the International Commission on Microbiological Specifications for Foods (ICMSF 2018) as risk category IIIB, which includes severe hazards for restricted populations, posing risks of death, chronic sequelae, or long‐term effects. Members of this genus have been implicated in uncommon yet life‐threatening diseases affecting neonates and infants, including necrotizing enterocolitis, meningitis, and sepsis. In addition, infections have been reported among elderly individuals and patients with compromised immune systems, encompassing conditions such as septicemia, pneumonia, osteomyelitis, splenic abscesses, and various wound‐related infections (Berthold‐Pluta et al. 2021). Accurate detection of these microorganisms remains difficult, largely because many laboratories do not have access to fast and reliable diagnostic techniques, which contributes to the underreporting of Cronobacter infections (Holý and Forsythe 2014). The *Cronobacter* genus comprises seven species: *Cronobacter* *sakazakii*, *Cronobacter* *turicensis*, *Cronobacter* *malonaticus*, *Cronobacter* *condimenti*, *Cronobacter* *dublinensis* (subsp. *dublinensis*, *lactaridi*, *lausannensis*), *Cronobacter* *muytjensii*, and *Cronobacter* *universalis*. Its high resistance to desiccation is most likely due to the trehalose content in *Cronobacter* cells, particularly in the stationary growth phase, acting as a protective factor (Feeney et al. 2014).

*Escherichia hermannii* was initially reported in 1982 and later recognized as a separate species within the *Escherichia* genus following the identification of biochemical and genomic distinctions from *Escherichia coli*. Although human infections attributed to this microorganism are uncommon and it is often considered a co‐pathogen rather than a primary etiological agent, available evidence indicates that it possesses sufficient virulence to induce sepsis in a manner comparable to *E. coli*. The clinical manifestations most frequently associated with *E. hermannii* include bacteremia, infections of the urinary tract, and involvement of the central nervous system (Ioannou 2019).

The genus *Mixta* was formally proposed in 2018 as a result of the taxonomic reassignment of four species previously classified within *Pantoea*, namely *Mixta calida*, *Mixta gaviniae*, *Mixta intestinalis*, and *Mixta theicola*. Both *M. gaviniae* and *M. calida* were initially recovered from powdered infant formula and from environments involved in its manufacture (Xia et al. 2020). *M. calida*, formerly designated *Pantoea calida*, is primarily an environmental microorganism and is only infrequently linked to human pathology. Nevertheless, isolated clinical reports have documented infections such as sepsis, meningitis, and cases involving implantable cardioverter‐defibrillators (Blairon et al. 2024). Recently, the literature reported a case of bacteremia and meningitis caused by *M. calida* in a 5‐week‐old infant, successfully treated with cefotaxime, a *β*‐lactam antibiotic (Van Hees et al. 2024). The microorganism *Kosakonia cowanii* is considered a rare pathogen that can promote acute cholecystitis, an infection of the gallbladder (Berinson et al. 2020).

In the present study, *Salmonella* sp. was detected in sample S11, which originated from the state of Goiás, the largest producer of baru nuts. Under conditions of reduced aw, *Salmonella* adapts by synthesizing inhibitors of cell division and by accumulating compatible solutes, including trehalose, which help preserve cellular turgor and osmotic balance. In addition, the high lipid content of the nuts provides a protective function for *Salmonella*, shielding it from gastric acid and thereby facilitating intestinal colonization, even at extremely low microbial loads. It is known that stress conditions caused by low aw can trigger changes in gene expression and protein synthesis as a defense mechanism. This allows microorganisms to endure adverse conditions, resulting in more virulent strains (Nascimento et al. 2018; Hertwig et al. 2019).

The spoilage bacterium *Rahnella inusitata* has been associated with post‐processing contamination of pasteurized fluid milk, resulting in a fibrous defect primarily linked to extracellular polysaccharides (Prinčič et al. 2024). Although the genus *Erwinia* is classically described as a phytopathogen, several studies identify it as a spoilage microorganism in plant‐based foods, especially vegetables and minimally processed products. It is not considered a typical human pathogen, but its presence is relevant as an indicator of quality or environmental contamination (Tournas 2005). It is important to highlight that *Erwinia* sp. can be explored for biotechnological applications, such as the production of the enzyme L‐asparaginase (Castro et al. 2021) and the synthesis of isomaltulose (Souza et al. 2022).

*Enterobacter hormaechei* microorganisms have been identified in studies as food contaminants, especially in fresh vegetables and processed food products. Their presence is concerning due to their ability to produce extended‐spectrum *β*‐lactamases (ESBLs) and their resistance to multiple antibiotics, representing a public health risk, particularly for vulnerable populations. One study isolated a MDR strain of *E. hormaechei* i from mixed sprouts, identifying resistance genes. The strain exhibited resistance to antibiotics such as cefotaxime, ceftazidime, tetracycline, and gentamicin, highlighting the risk of resistance transmission through the consumption of contaminated vegetables (Leighton et al. 2024).

Previous studies have documented the recovery of colistin‐resistant *E. hormaechei* from bulk raw milk. The detection of colistin‐resistant members of the *Enterobacteriaceae* family in food matrices represents a growing public health concern, as these organisms may act as reservoirs for resistance traits transferable to human pathogens. The authors highlight the importance of implementing molecular‐based diagnostic tools to enable rapid and precise identification of resistance genes, which is fundamental for the mitigation and management of MDR infections and for improving the understanding of the epidemiological and dissemination patterns of antimicrobial resistance (AMR). Moreover, the integration of genomic approaches with conventional phenotypic susceptibility testing allows a more complete characterization of resistance mechanisms, thereby supporting informed therapeutic decision‐making. Addressing the expansion of antibiotic resistance also requires increased investment in the development of robust prevention and control strategies (Fraccalvieri et al. 2025).

To the best of current knowledge, this is the first report of the isolation of *Metakosakonia massiliensis* from a food matrix, specifically from baru nuts. The presence of this species in a plant‐based food suggests a possible environmental origin or contamination during processing, representing a novel finding that expands the understanding of the microbial ecology of this genus.

From a One Health perspective, the presence of diverse members of the *Enterobacteriaceae* in baru nuts highlights the complex interactions between environmental reservoirs, food production systems, and human health. These microorganisms are widely distributed in soil, water, plants, animals, and the broader environment, and their occurrence in food products may reflect multiple contamination pathways throughout the production chain. In addition, the detection of pathogens and species associated with AMR reinforces the importance of integrated surveillance strategies that consider environmental, agricultural, and public health dimensions simultaneously. Understanding these interconnected pathways is essential for improving risk assessment and for developing preventive measures that protect both ecosystem and human health.

## 4 Conclusion

Brazil is a key exporter of native nuts, significantly contributing to regional development and the bioeconomy. Baru nuts, which are increasingly gaining space in international markets, face challenges related to the control of spoilage and pathogenic microorganisms in order to extend shelf life and ensure microbiological quality. Despite the limited availability of rapid diagnostic tools and the still scarce knowledge regarding the microbial diversity associated with edible nuts such as baru, this study showed that *S. aureus*, lactic acid bacteria, and thermophilic spore‐formers were below quantification limits, whereas mesophilic aerobic spore‐formers, total aerobic mesophiles, *Enterobacteriaceae*, molds, and yeasts were detected at varying levels among samples. The identification of potentially pathogenic and MDR species further highlights the need for continuous microbiological surveillance and the rigorous implementation of good agricultural and processing practices throughout the production chain. In this context, the findings of this study may support the development of risk‐based monitoring strategies and contribute to food safety management, regulatory oversight, and compliance with microbiological standards required in international markets. Furthermore, these data may assist epidemiological investigations and microbial risk assessments, advancing the understanding of the microbial ecology of baru nuts and contributing to the safety and quality of this increasingly valued product.

Future studies should further investigate the sources and dynamics of microbial contamination along the baru nut production chain, as well as the genomic characterization of pathogenic isolates, including the potential occurrence of AMR determinants, stress response genes, and virulence factors. The application of whole‐genome sequencing (WGS) approaches may provide deeper insights into the genetic profiles of foodborne pathogens, such as *Salmonella* and *Cronobacter*, contributing to a better understanding of their adaptation and persistence in food systems. Such knowledge can provide a scientific basis for the development of targeted prevention and control strategies aimed at improving the microbiological safety of baru nuts and other edible nuts.

## Author Contributions

**Clara Mariana Gonçalves Lima**: conceptualization, formal analysis, investigation, writing – original draft, writing – review and editing, methodology, visualization, data curation. **Dionísio Pedro Amorim‐neto**: investigation, writing – original draft, visualization, validation, methodology, formal analysis. **Bruna Godoi de Castro**: investigation, writing – original draft, visualization, validation, methodology. **Ana Luísa Perini Leme Giordano**: investigation, writing – original draft, visualization, validation, methodology, formal analysis. **Beatriz Machado Dal Pian**: investigation, writing – original draft, validation, visualization, methodology. **Matheus Péricles Silva Láscaris**: investigation, writing – original draft, visualization, validation, methodology. **Luana Cristina da Silva Ramos**: investigation, writing – original draft, visualization, validation, methodology. **Angélica Zaninelli Schreiber**: investigation, writing – original draft, funding acquisition, validation, visualization, methodology, formal analysis, supervision, resources. **Luisa Freire Colombo**: investigation, writing – original draft, visualization, validation, methodology, resources, supervision. **Anderson S. Sant'Ana**: conceptualization, investigation, funding acquisition, writing – original draft, writing – review and editing, visualization, validation, methodology, project administration, supervision, resources.

## Conflicts of Interest

The authors declare no conflicts of interest.
